# Nerve regeneration using decellularized tissues: challenges and opportunities

**DOI:** 10.3389/fnins.2023.1295563

**Published:** 2023-10-19

**Authors:** Maryam Mahdian, Tayebeh Sadat Tabatabai, Zahra Abpeikar, Leila Rezakhani, Mozafar Khazaei

**Affiliations:** ^1^Student Research Committee, Kermanshah University of Medical Sciences, Kermanshah, Iran; ^2^Student Research Committee, School of Medicine, Shahroud University of Medical Sciences, Shahroud, Iran; ^3^Department of Tissue Engineering, School of Advanced Technologies in Medicine, Fasa University of Medical Sciences, Fasa, Iran; ^4^Fertility and Infertility Research Center, Health Technology Institute, Kermanshah University of Medical Sciences, Kermanshah, Iran; ^5^Department of Tissue Engineering, School of Medicine, Kermanshah University of Medical Sciences, Kermanshah, Iran

**Keywords:** nerve regeneration, decellularized tissue, PNS, CNS, extracellular matrix

## Abstract

In tissue engineering, the decellularization of organs and tissues as a biological scaffold plays a critical role in the repair of neurodegenerative diseases. Various protocols for cell removal can distinguish the effects of treatment ability, tissue structure, and extracellular matrix (ECM) ability. Despite considerable progress in nerve regeneration and functional recovery, the slow regeneration and recovery potential of the central nervous system (CNS) remains a challenge. The success of neural tissue engineering is primarily influenced by composition, microstructure, and mechanical properties. The primary objective of restorative techniques is to guide existing axons properly toward the distal end of the damaged nerve and the target organs. However, due to the limitations of nerve autografts, researchers are seeking alternative methods with high therapeutic efficiency and without the limitations of autograft transplantation. Decellularization scaffolds, due to their lack of immunogenicity and the preservation of essential factors in the ECM and high angiogenic ability, provide a suitable three-dimensional (3D) substrate for the adhesion and growth of axons being repaired toward the target organs. This study focuses on mentioning the types of scaffolds used in nerve regeneration, and the methods of tissue decellularization, and specifically explores the use of decellularized nerve tissues (DNT) for nerve transplantation.

## Introduction

The central nervous system (CNS) includes the nervous tissue of the brain and spinal cord, and diseases such as multiple sclerosis, stroke, Alzheimer’s, Parkinson’s, and Huntington’s disease cause disorders in this system. Peripheral nervous system (PNS) refers to nerve bundles outside of the CNS and its disorders include Guillain-Barre syndrome, peripheral neuropathy, and radiculopathy (Borsook, 2012). Although no long-lasting treatments exist for neurological illnesses, especially in the CNS, cell therapy offers a potential way to repair degenerative tissue. Based on biomaterials, tissue engineering holds promise for neuro-regeneration and repair (Akhtar et al., 2022). This can be achieved using embryonic or fetal cells, but ethical concerns and a high likelihood of tissue rejection are associated with this approach. On the other hand, using adult cells like neural stem cells allows for autologous grafts with fewer ethical issues (Sivandzade and Cucullo, 2021; Sharifi et al., 2022).

In the field of neurological regeneration, the use of decellularized tissues through various physical and chemical protocols has shown significant promise in both animal and human clinical applications (Teebken et al., 2000). The tissues obtained from the normal nerve structure and extracellular matrix (ECM) can be transplanted as graft tissues (Gilbert et al., 2006; Nune et al., 2016). This review article specifically focuses on nerve regeneration using decellularized nerve tissues (DNT). In recent studies, the use of biological scaffolds has been the focus of researchers in nerve repair (Hsu et al., 2023; Yu et al., 2023). Therefore, we decided to mention this issue in this paper, and we hope that we have helped those interested in this field.

**Figure 1:**
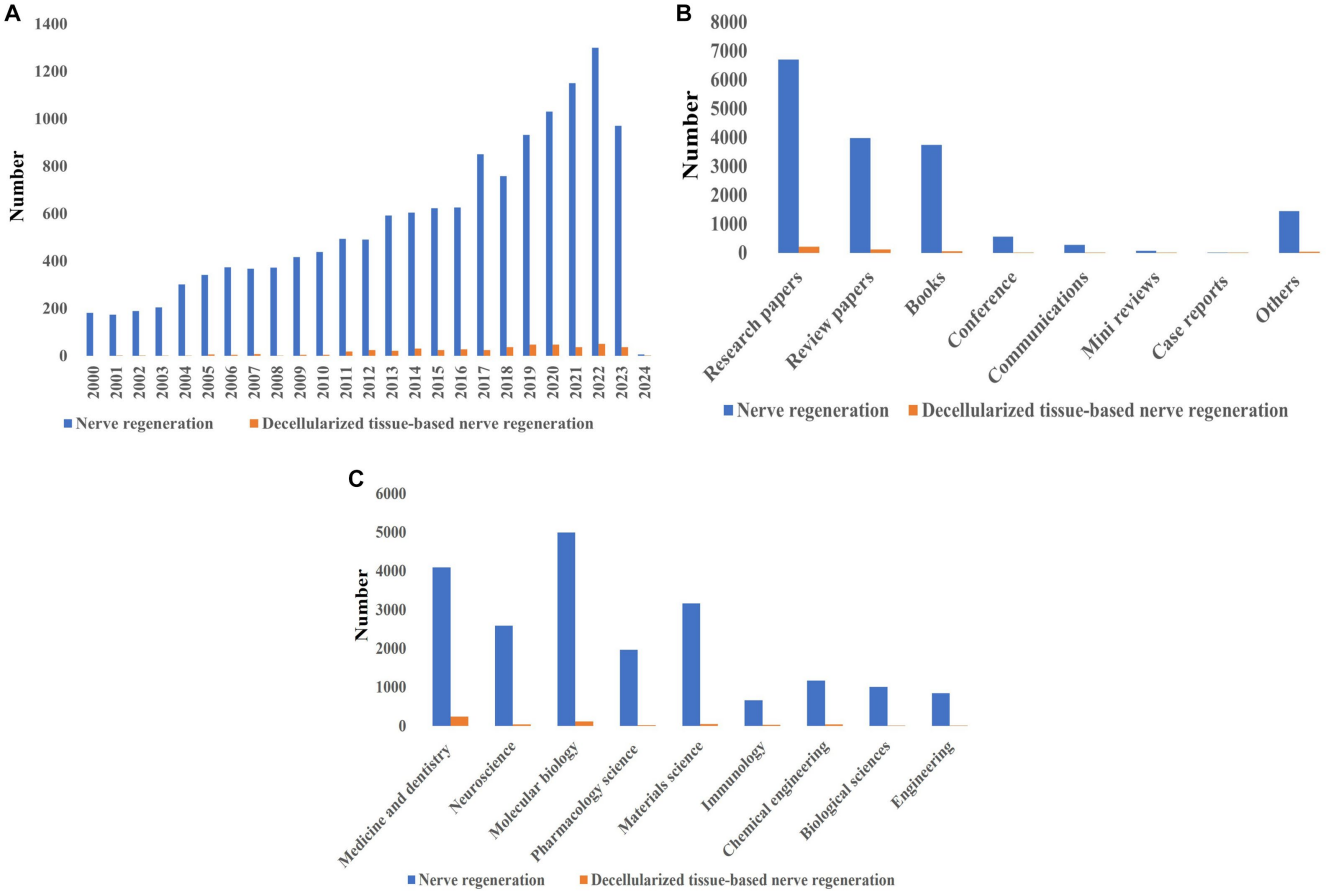
Scientific attention to nerve regeneration based on Scopus, PubMed, and Web of Science reports: **(A)** Number of published articles on nerve regeneration with and without DNT from 2000 to 2024, **(B)** type of published literature on nerve regeneration with and without DNT, **(C)** statistics of scientific attention to different branches of nerve regeneration with and without DNT.

Figure 1 illustrates the history of nerve regeneration with and without DNT, along with the number of related articles and their research field. Nerve regeneration is a topic that has been receiving the attention of medical science for many years, and the process of nerve grafting using different materials has been researched over time. But recently, researchers’ attention has grown to repair damaged nerves using decellularized tissues, which has led to the publication of numerous articles and books and the holding of various scientific conferences in this field. The use of decellularized tissues as the new approaches in nerve transplantation, especially in different scientific branches, has been analyzed due to its unique features. This innovative technique can help repair and regenerate nerves over time. Therefore, all these efforts and developments can be significantly effective in advancing science in the field of nerve regeneration.

## CNS/PNS injury

CNS traumatic injuries, with their destructive and persistent complications, include traumatic brain injury (TBI) and traumatic spinal cord injury (SCI) (Roozenbeek et al., 2013; Walter and Zweckberger, 2018). TBI encompasses various neurological disorders such as tissue contusion, subdural and epidural hematoma, penetrating injuries, or diffuse damage, and can be classified by the Glasgow Coma Scale (GCS) into severe [3–8], moderate [9–12], and mild [13–15] (Maas et al., 2008). Although there is no precise analysis of physical function, determining the exact location and size of the lesion is significant for assessing the extent of functional recovery after the injury (Fouad et al., 2021).

Wallerian degeneration (WD) is a process that occurs in both CNS and PNS traumatic injuries, as well as in neurodegenerative diseases such as Alzheimer’s disease (AD) and Parkinson’s disease (PD), leading to degeneration of distal axons detached from their cell bodies (Rotshenker, 2011). Furthermore, there are contradictory findings regarding the effect of inflammation after CNS trauma on neurons; some studies show a detrimental effect, while others suggest it stimulates axon regeneration (Yin et al., 2006). The pathophysiology of SCI includes the death of neural cells and disruption of axonal connections. This degenerative process in distal long axons is called WD, but in the proximal stem, it can lead to the vulnerability of cell bodies (Ruff et al., 2008).

**Figure 2:**
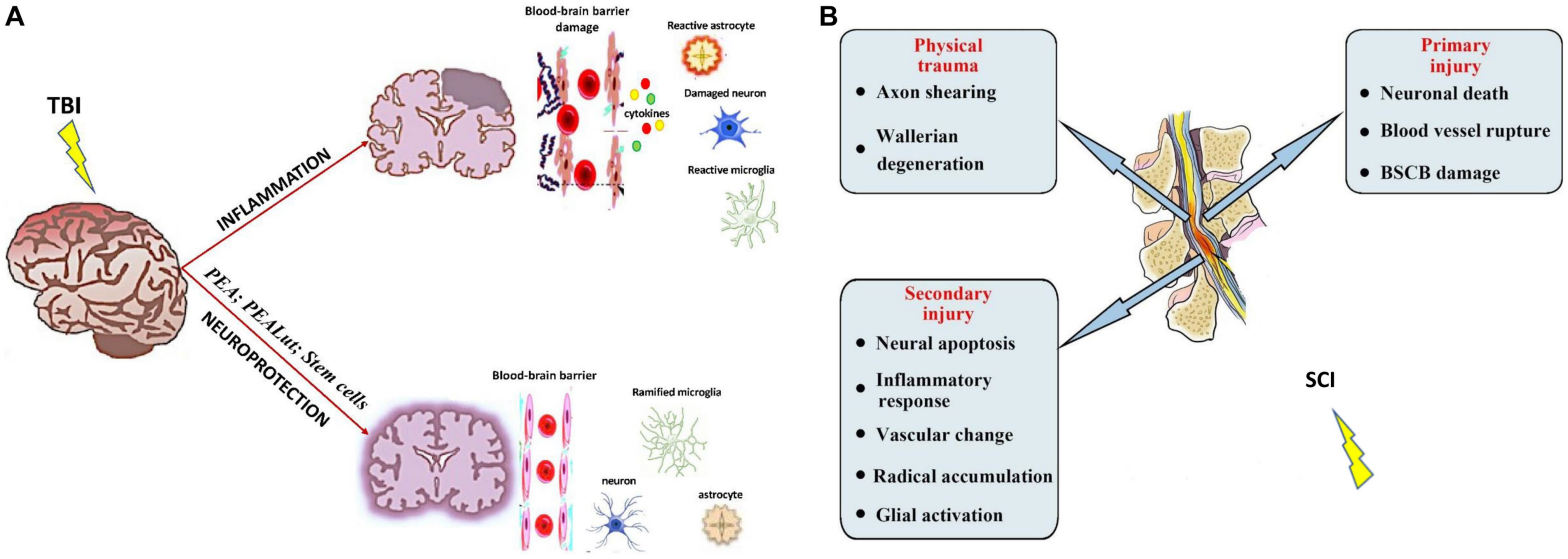
TBI **(A)** (Crupi et al., 2020) and SCI **(B)** (Feng et al., 2021) are serious neurological conditions, each with its pathophysiological processes following the injury. Adapted and reprinted.

The PNS is composed of neuronal cells, glial cells, and stromal cells that conduct signals between the CNS and the body (Menorca et al., 2013). Challenges resulting from PNS injury include focal demyelination (neurapraxia), axon damage in addition to focal demyelination with normal connective tissue (axonotmesis), and complete dissection of axons and connective tissue (neurotmesis). Additionally, inflammation in the PNS plays a positive role in axon regeneration, but the precise mechanism of this immune reaction is not fully understood (Ankeny et al., 2009; Figure 2).

## Nerve repair with tissue engineering scaffolds

The treatment of PNS, particularly in the crucial gap of more than 3 cm, remains a significant clinical issue despite the tremendous advancements in medicine (Hussin et al., 2018). According to reports, many variables, such as the extent of the injury, the presence of neurotrophic factors, the existence of Schwann cells, etc., affect the restricted regeneration of peripheral nerves. Peripheral nerve damage is one of the most frequent causes of sensorimotor abnormalities and decreased productivity in adults, according to the literature (Moore et al., 2011). Since peripheral nerves have a limited capacity for self-regeneration, tissue engineering is employed as a potential method for nerve repair. The defect and formation of a gap in the peripheral nerve result in a severe loss of sensory or motor function in the affected animal or person. When the damage is minor, the nervous system may mend itself, which is a complicated biological event. It is challenging to restore the injured nerve in severe injuries that result in a large gap (Gontika et al., 2018).

Stem cells, natural and man-made materials, decellularized grafts, and other cell types are frequently utilized in regenerative medicine, a large branch of medical sciences, to restore tissues or damaged organs (Campbell, 2008). The creation and restoration of an organ’s normal function are the ultimate objectives of regenerative medicine. Tissue engineers have created innovative compounds and scaffolds of organic and synthetic materials with physical, chemical, and biological characteristics, targeting tissues. The creation of nerve conduits results from researchers’ efforts to address the drawbacks of nerve autograft transplantation (Yu et al., 2023).

To find a solution for the reconstruction and transplantation of organs, xenografts were proposed, but they were also rejected. The existing limitations for transplantation have led to the use of gene-edited pigs as an effective source for this purpose. The creation of pigs devoid of xenoantigens has been made possible via nuclease-based genome alteration techniques combined with somatic cell nuclear transfer (SCNT). These pigs’ organs prevent hyperacute rejection (HAR) and last longer after being transplanted into non-human primates (NHPs) (Navarro-Serna et al., 2022). A clinical xenotransplantation experiment using porcine skin grafts from pigs was launched in 2019 with FDA approval. At the end of the 30-day experimental period, preliminary findings indicated satisfactory graft acceptance and there was no indication of zoonotic disease transmission (Eyestone et al., 2020). These efforts have led to the development of several efficient xenotransplantation techniques.

Currently, both natural and artificial materials, including both autologous and non-autologous tissue transplants, and tissue, and cell transplants, are employed to regenerate neural tissues. End-to-end repair is the main therapy used to get rid of these lesions. Peripheral nerve grafts or nerve conduits will be employed if end-to-end repair is not possible (Jiang et al., 2023).

### Neural conduits made of synthetic and natural polymers

Recent advancements in nerve conduit technology have utilized a combination of synthetic and natural polymers, such as Oligo-polyethylene glycol fumarate (OPF), Poly caprolactone (PCL), Poly-L-lactic acid (PLLA), Polycaprolactone fumarate (PCLF), Polylactic -co-glycolic acid (PLGA), collagen, chitosan, and fibrin, to facilitate nerve regeneration (Liu et al., 2017). Engineered nerve conduits, resembling nerve tissue with a collagen basis, encourage axon rebuilding, inhibit scar tissue invasion, and enhance the production of neurotrophic factors (Daly et al., 2013).

Bioactive properties in proteins (silk, collagen, etc.), polysaccharides (hyaluronic acid, chitosan, etc.), and polyesters (P3HB and PHBV) improve cell-scaffold interaction, facilitating tissue regeneration (Arslantunali et al., 2014; Ehterami et al., 2022). Biodegradable polymeric nerve conduits based on copolymers like poly-L lactide, polyurethane, and polyglycolic acid show promise as ideal scaffolds due to their bio-absorbability and mechanical characteristics (Cingolani et al., 2018; Ehterami et al., 2021).

These thermoplastic materials can be produced through various methods such as extruding, molding, printing, and dipping (Li et al., 2020). Natural polymers, when combined with synthetic polymers like PCL with collagen-based beads, exhibit enhanced strength and flexibility. Several neural conduits made from synthetic and natural polymers have received: food and drug administration (FDA) approval for clinical use (Deal et al., 2012, Kehoe et al., 2012). While artificial nerve conduits have some drawbacks, including inflammation and stiffness, biological conduits made of veins, arteries, and fibroblasts show promise (Hussin et al., 2018). Natural biological nerve conduits, including vessels, decellularized nerves, and muscle tissue, have been successfully used in animal models and human practice, offering a favorable environment for cell adhesion and proliferation, promoting axon regeneration and vascularization (Gontika et al., 2018; Table 1).

**Table 1:** The most significant natural and synthetic polymers utilized in nerve tissue engineering.

Polymer	Source	Property	References
Collagen	Natural	Collagen, which has a triple helix structure, a smooth microgeometry, and high permeability (approximately D 100,000), is the primary component of the extracellular matrix.	Yu et al. (2011), Song et al. (2016), Fertala et al. (2020), and Yuan et al. (2023)
Chitosan	Natural	Chitosan, a versatile polymer derived from glucosamine and N-acetyl glucosamine, offers biocompatibility, biodegradability, non-toxicity, affordability, and adaptability for tissue regeneration in various forms like films, scaffolds, hydrogels, and membranes.	Wang et al. (2005)
CMC	Natural	Carboxymethylchitosan: Amphoteric polyelectrolyte, OH/NH2/COOH groups, antibacterial, antioxidant, apoptosis inhibitor.	Zhang et al. (2022b)
PLCL	Synthetic	High flexibility, rapid decomposition, and low glass transition temperature (approximately −60) are all characteristics of the semi-crystalline polymer known as PLCL.	Bachtiar et al. (2021)
PHAS	Natural	This material is a biodegradable, biocompatible, and thermoplastic biological polyester.	Williams et al. (1999)
PHB	Natural	PHB is a bacterial storage product that may be converted into sheets, particles, and absorbent films. It also has the capacity to increase the number of Schwann cells.	Hazari et al. (1999)
PHBV	Natural	Among the qualities of PHBV are its capacity to modify physical properties, process them in various ways, increase flexibility, and lower the glass transition and melting temperatures in copolymer structures.	Biazar and Heidari Keshel (2013)
Gelatin	Natural	This denatured collagen-based polymer accelerates nerve transmission, boosts axon quantity, and prevents internal connective tissue growth in nerve conduits.	Chen et al. (2005)
Fibronectin	Natural	This disulfide glycoprotein, one of the key components of the ECM, has the power to improve cell adherence, morphology, migration, and neural development.	Ahmed and Brown (1999)
SF	Natural	A fibrous protein from worm and butterfly glands, it’s highly elastic, mechanically strong, with a high modulus, and biocompatible and biodegradable.	Schacht and Scheibel (2014)
Creatine	Natural	This polymer can induce nerves.	Sierpinski et al. (2008)
HA	Natural	Hyaluronic acid (HA) is a biocompatible polymer with high water retention, low immunogenicity, and the ability to bind to specific cell surface receptors. It can take various forms, including viscoelastic solutions, hydrogels, sheets, and nanoparticles, and enhances progenitor cell and NSC adhesion due to its unique properties.	Burdick and Prestwich (2011)
PLA	Synthetic	This polymer, derived from lactic acid (from sources like sugar beet, corn, or wheat), offers biocompatibility, thermoplasticity, strong mechanical resistance, excellent biodegradability, and the ability to accelerate regeneration and functional recovery in sciatic nerve injuries.	Zeng et al. (2015) and Mao et al. (2023)
PLLA	Synthetic	The conduits created by PLLA are extremely porous with an interconnected pore structure and have a regular and highly crystalline.	Evans et al. (1999)
PGA	Synthetic	It belongs to a class of polyesters that are rigid, thermoplastic, highly crystalline, have a high tensile modulus, and have very little solubility in organic solvents.	Lee et al. (2017) and Li et al. (2023)
PLGA	Synthetic	It is a co-polyester that is simple to manufacture, has a minimal inflammatory reaction, and can adapt for axonal growth and nervous system regeneration.	Mir et al. (2017)
PCL	Synthetic	A polyester that is highly soluble in organic solvents, has a glass transition temperature of about (−60), and has a low melting temperature of 55 to 60.	Bolaina-Lorenzo et al. (2016) and Lu et al. (2023)
PU	Synthetic	Many medical devices, including nerve conduits, are made using a polymer having a structure made of urethane linkages. Additionally, this polymer can regrow myelinated neurons.	Gautam et al. (2007)
PVA	Synthetic	The properties of this polymer include a biocompatible crystalline structure, solubility in water, non-degradability, non-toxicity on axon formation, biodegradability, hydrophilic nature, and swelling ability.	Gaaz et al. (2015)
HYAFF	Synthetic	It is one of the hyaluronic acid derivatives that is created when hyaluronic acid is esterified with benzyl ester. One of the characteristics of this polymer is its capacity to process in many forms while also promoting the proliferation of various cell types.	Wang et al. (2012)
PPY	Synthetic	Among this polymer’s characteristics are its capacity to promote cell adhesion, biodegradability, conductivity, insolubility, poor stability, non-allergenicity, non-mutagenicity, and insolubility.	Liu et al. (2015)
PANI	Synthetic	Conductivity, the capacity to promote adhesion, proliferating cells, and neurite development are only a few of this polymer’s attributes.	Alhosseini et al. (2012)
PEDOT	Synthetic	This polymer contains characteristics like conductivity, and the capacity to promote adhesion, proliferation, and neurite development.	Xie et al. (2009)
CNT	Synthetic	Among the properties of this polymer are conductivity, inherent pressure absorption capacity, induction of conductivity, the capacity to promote adhesion, and the capacity to lengthen dendrites.	Huang et al. (2010) and Pi et al. (2022)
Lignin	Synthetic	This polymer is recognized for its branched structure, molecular mass, high carbon content, exceptional hardness, low density, antioxidant activity, and biodegradability.	Amini et al. (2021)
Silicon	Synthetic	This polymer’s attributes include biocompatibility, flexibility, and accessibility in a variety of diameters.	Ozdil and Aydin (2014)
Albumin	Natural	This water-soluble protein, making up around 50% of blood plasma mass, transports fatty acids and regulates blood pH. It has biocompatibility, biodegradability, weak mechanical properties, and can be formed into nanoparticles, microparticles, and fibers.	Eschbach (2000)
PEEK	Synthetic	A linear, aliphatic, semi-crystalline polymer with a melting point of 334 and good wear resistance, PEEK has high efficiency. Its Young’s modulus is around 3.7 Gpa, and its maximum tensile strength is 100 MPa.	Eschbach (2000)

## Extracellular matrix

ECM consists of proteoglycans, fibronectin, vitronectin, laminin, tenascin, thrombospondin, fibrillin, and collagen, which play a crucial role in adherence, duplication, and differentiation functions (Badylak et al., 2015). Any disruption in the regular arrangement of these biomolecules can lead to various diseases (Hinderer et al., 2016).

As we all know, tissue engineering efficiently regenerates damaged tissues using a mix of cells, scaffolds, and growth factors. Unfortunately, designed scaffolds of non-native materials frequently fail to establish adequate cell connections, which prevents essential functions from occurring during tissue healing. Using natural ECMs with various functional properties, such as structural support for cells, provision of a mechanical environment for cells, biodegradability to form functional microvascular networks, biocompatibility, and appropriate biological activities, can help overcome these difficulties (O'brien, 2011). Therefore, a scaffold matching the target tissue’s original ECM architecture would be optimal for regenerative medicine. However, given the present technology, effective natural ECM mimicking for synthetic tissues continues to be difficult (Kim et al., 2016).

Unfortunately, there are several obstacles in the way of tissue-derived ECM’s therapeutic use. ECM made from animal tissue is used as an alternate source due to the restricted availability of human cadaveric tissue. Immune rejection and disease spread are possible with incomplete tissue decellularization. Due to the difficulty in isolating some specialized tissues, including stem cell niches, some ECMs are not readily accessible. Due to these reasons, not all tissue regeneration procedures may employ ECM produced from decellularized tissues (Assunção et al., 2020). In particular, neural ECMs enable neural stem cell differentiation and promote axonal development *in vitro*, which have demonstrated positive outcomes for several neural tissue regeneration issues such as spinal cord injuries. The decellularized swine brain in mice with spinal cord injury (SCI) restored motor function up to two months after injury and increased the reparative macrophage phenotype. It’s interesting to note that optic nerve decellularization produced the best possible optic nerve performance by removing some axonal growth inhibitory molecules while leaving others intact (Ma et al., 2021; da Silva et al., 2023).

## Definition and methods of tissue decellularization

**Figure 3:**
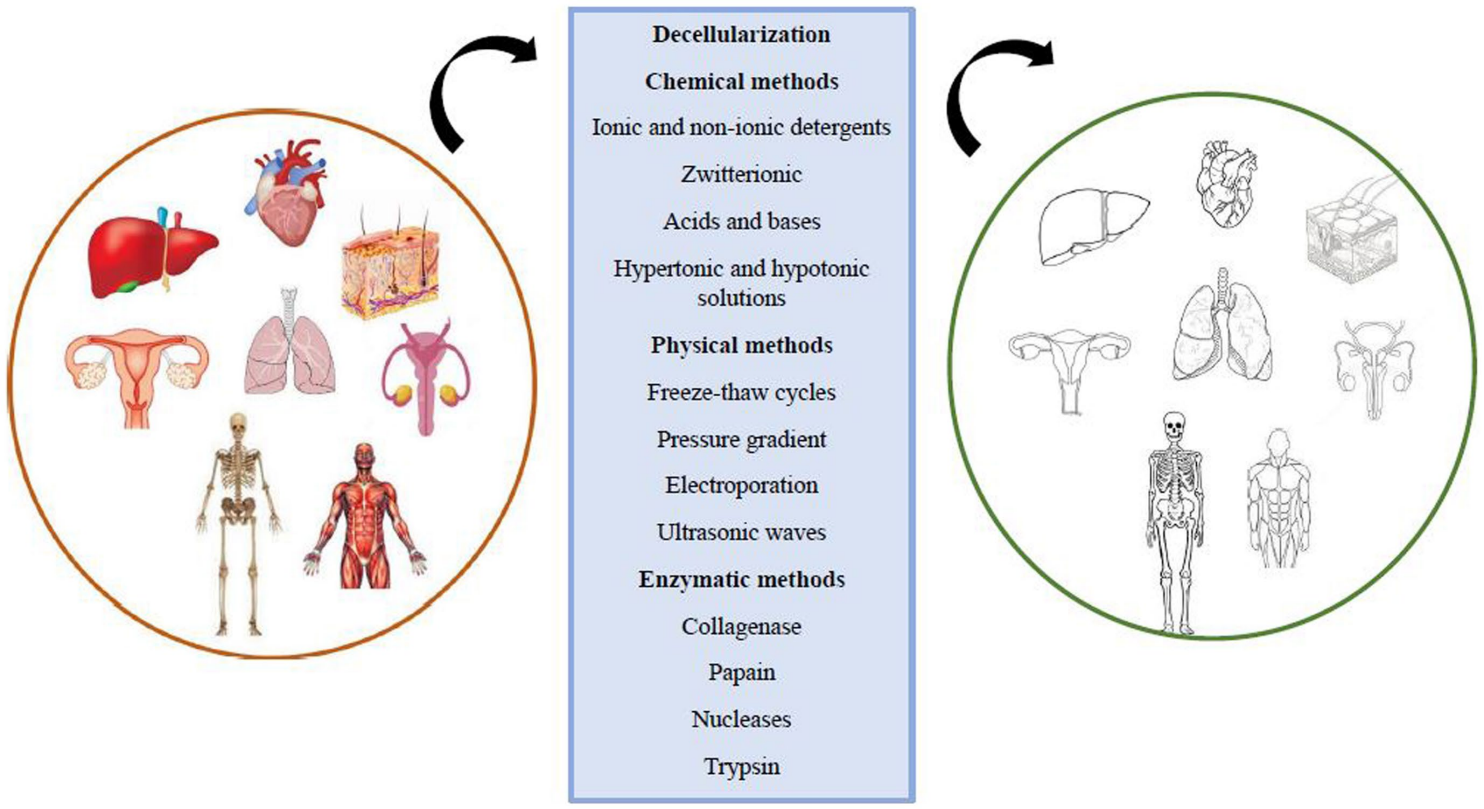
Common methods of tissue decellularization.

Scaffolds are crucial in tissue engineering as they facilitate the attachment and growth of foreign cells, providing mechanical and structural stability while distributing necessary growth factors for tissue regeneration. Scaffolds can be made from synthetic materials or natural tissues, with natural scaffolds offering biocompatibility and encouraging proper cellular interactions. Decellularization is a process that removes cellular components from the extracellular matrix (ECM) while preserving its structural and functional proteins and glycosaminoglycans. This creates a natural 3D form with regenerative properties (Mendibil et al., 2020). This comment is mentioned in the nerve repair with tissue engineering scaffolds section (Alizadeh et al., 2020). Decellularization eliminates the risk of inflammatory reactions and immunological rejection at the transplant site by removing cellular components and antigens. However, striking a balance between retaining the ECM structure and removing cellular waste products is essential to prevent inflammatory responses and support healthy regeneration. The ECM composition, surface topology, and 3D structure play crucial roles in host tissue regeneration responses, cell migration, proliferation, differentiation, mitogenesis, and chemotaxis. Decellularization can be applied to various tissues using physical, chemical, and enzymatic methods, or their combinations (Alizadeh et al., 2021; Khazaei et al., 2022) (Figure 3).

### Chemical techniques

Chemical detergents, acids, bases, alcohols, and hypotonic and hypertonic solutions are used in decellularization processes. Detergents are polar and hydrophobic amphipathic molecules that can dissolve hydrophobic substances in water. Based on their polar head, detergents are categorized into three groups: ionic, non-ionic, and zwitterionic. Detergents that are ionic, non-ionic, or zwitterionic effectively remove biological components from the tissue by separating DNA from proteins and cell membranes. However, these elements disrupt the ECM’s protein structure (Zahmati et al., 2017; Khazaei et al., 2023).

Ionic detergents: Ionic detergents are potent cleaning agents that may fully denature proteins and obliterate cell membranes. Among the most used ionic decellularization agents are sodium hypochlorite, sodium deoxycholate (SDC), and sodium dodecyl sulfate (SDS). Cytoplasmic membranes, lipids, and DNA may all be successfully dissolved by these detergents. The process of decellularization frequently employs SDS (Montoya and McFetridge, 2009). SDS is more efficient than other detergents at removing cell waste products like nuclear and cytoplasmic substances from dense and thick tissues like the heart. As a result, it is the detergent that is employed in the decellularization process the most frequently. 1%SDS (w/v) is the most typical concentration in the decellularization procedure. SDS breaks down the covalent links that hold proteins together (Syazwani et al., 2015). Non-ionic detergents: less damaging to the ECM’s structural integrity. However, the decellularization process makes extensive use of these detergents. Protein–protein bindings cannot be broken by non-ionic detergents, however lipid-lipid and lipid-protein bonds can be (Alizadeh et al., 2022).

Decellularization uses Triton X-100 as a non-ionic detergent (El-Husseiny et al., 2023). When decellularizing thin tissues, zwitterionic or bipolar detergents—which have both ionic and nonionic properties—are frequently employed. Proteins are protected in their native condition throughout the decellularization process by zwitterionic detergents’ hydrophilic groups, which have zero net electric charges. These cleaners include: (3-cholamidopropyl) (dimethylammonio]-1-propane sulfonate (CHAPS), Sulfobetaine 16 (SB-16) and Sulfobetaine 16 (SB-16), for instance (Faulk et al., 2014).

Acids and bases: Acids and bases are catalysts or agents that hydrolyze biomolecules. Chromosomes and plasmid DNA are disrupted by bases. Ammonium hydroxide, sodium sulfide, sodium hydroxide, and calcium hydroxide are examples of bases (Reing et al., 2010). During the earliest steps of decellularization, the hair from dermis samples is typically removed using sufficiently coarse blades. Collagen fiber degradation and collagen cross-link disruption are the main mechanisms through which bases impair mechanical characteristics (Gupta et al., 2018). By dissolving cytoplasmic substances and rupturing nucleic acids, acids operate to remove DNA from ECM. They can also denature ECM proteins such as glycosaminoglycans (GAGs), collagen, and growth factors (Bourgine et al., 2013). Acetic acid, peracetic acid, hydrochloric acid, and sulfuric acid are among the acids employed in decellularization procedures. It should be mentioned that it’s crucial to optimize both the dose and the timing of the acids employed for decellularization (Gilbert, 2012).

Alcohols: By lysing and drying out cells, alcohols like glycerol aid in the decellularization process. According to reports, alcohol can be used to tackle the problem of phospholipids causing calcification and prosthesis failure in valves and conduits (Montoya and McFetridge, 2009). Alcohols like isopropanol, ethanol, and methanol may turn a tissue into one that is free of fat in a very short amount of time and is far more efficient than lipases in doing so. A crucial factor is the need for extreme caution while decellularizing tissues with alcohol. Because alcohols like ethanol and methanol may fix tissue, precipitate proteins, and harm the ECM’s structure. Additionally, as a last wash to get rid of any remaining nucleic acid in the tissue, ethanol or methanol might be utilized (Somuncu, 2020).

Solutions that are either hypertonic or too hypotonic lead to osmotic stress in the tissue and cell membrane breakdown in tissues or organs, which results in cell lysis. Proteins and DNA can be separated by hypertonic solutions. The washing of cellular debris is one of the purposes of utilizing hypertonic and hypotonic solutions, yet these solutions fall short of properly cleaning and removing the cellular debris from the tissues. Simple osmotic actions in hypotonic fluids can quickly lead to cell lysis with no alteration of the matrix molecules or their structural makeup. Tissues should be submerged alternately in hypertonic and hypotonic solutions to see the greatest osmotic effects. After the cells have been lysed, washing repeatedly with hypertonic and hypotonic liquids aids in washing the leftover cells from the tissue (Gilbert, 2012).

Chelators and Toxins: By binding to divalent cations, ethylene glycol, tetraacetic acid, and ethylene diamine tetraacetic acid functions as a chelating agent in the decellularization of organs. By separating ions like Ca^2+^ and Mg^2+^, the chemical components of these substances like ethylenediaminetetraacetic acid (EDTA) and ethylene glycol-bis (β-aminoethyl ether)-N, N,N′,N′-tetraacetic acid (EGTA) break the cell’s attachment to collagen and fibronectin. Chelating agents are typically employed in conjunction with other detergents or enzymes like trypsin since they are ineffective on their own (Goldstein and Thayer, 2014). Additionally, it has been mentioned that cytotoxic substances can be utilized in the decellularization of cells since toxins can harm cells (Keane et al., 2015).

### Physical techniques

Cells in tissue and organs are extensively destroyed by thermal shock and freezing and thawing cycles. However, some of the membrane and cell material are still present and can be eliminated using further decellularization techniques. Due to the geometrical form of the ice crystals, this approach may disrupt the cell scaffold and has little impact on the mechanical capabilities of the ECM. It also partially destroys the ECM structure (Khazaei et al., 2021). In this process, ice crystals fill the inside of the cell, causing the cell membrane to burst. The use of freezing and thawing cycles might lessen immunological reactions that are unfavorable, including leukocyte infiltration in vascular ECM scaffolds. The melting temperature (37°C) and the freezing temperature can be unilaterally altered in this method’s temperature range. Varied authors have employed varied and arbitrary numbers of thermal shock cycles (Poornejad et al., 2015).

Mechanical and hydrostatic pressure: By physically scraping the tissue or organ with the use of sharp or abrasive instruments with enzymes or salt solution, the surface cells of the tissue or organ can be successfully removed. Better decellularization of tissues and organs is facilitated by the physical removal of the surface layers, but the amount of force used must be exact since the membrane connections and underlying structure are sensitive to any form of direct mechanical stress (Santoso et al., 2014). In the hydrostatic pressure approach, the target tissue is sprayed with pressured water, which works faster and more effectively than the procedure that uses detergents or enzymes. However, the ECM structure might be harmed by the ice crystals that develop when there is water present. Therefore, using this approach to raise the temperature during the decellularization process eliminates the chance of ice crystal formation. However, this temperature rise also raises entropy, which makes the ECM more prone to failure (Watanabe et al., 2019).

Pressure gradient: Along with the use of enzymes, pressure gradient induction is a technique to aid in improved decellularization. The use of a pressure gradient offers the chance for more appropriate penetration of the enzyme agent into the tissue, and as a result, the tissue decellularization process will be carried out with a higher percentage in the decellularization of hollow tissues like veins or thicker tissues like tendons (Sierad et al., 2015).

Electroporation (permeabilizing the cell membrane): This technique might be cited among others for decellularizing tissues or organs. In this technique, the tissue is exposed to microsecond electrical pulses that cause the cell membrane to develop tiny holes. Cell homeostasis loss and eventual cell death are also possible outcomes of these holes. The relatively tiny electrodes used in this approach, which restrict tissue decellularization and make it unsuitable for vast tissues, are one of its drawbacks. More crucially, since the immune system will be responsible for the cell elimination mechanism, the decellularization process must be carried out inside the body. As a result, the applications of this technology are severely constrained (Sano et al., 2010).

Ultrasonic waves: Cells in a tissue or organ bath are separated using ultrasonic waves, which are frequently utilized at frequencies higher than 20 kHz. Cell membranes, internal components, and intermolecular connections can all be destroyed by these strong waves. The waves’ destructiveness increases with decreasing frequency (Azhim et al., 2011). Sonication refers to the use of ultrasonic waves for a variety of purposes. It’s crucial to keep bubbling under control while the procedure is going on. Due to the extreme fluid pressure fluctuations brought on by waves, this physical phenomenon is unavoidable, yet unchecked bubbling can seriously harm the tissue’s structure and mechanical capabilities. Additionally, the temperature, viscosity, and amount of gas dissolved in the various fluids all affect how much bubbling occurs (Forouzesh et al., 2019). Vacuum: The transmission of detergents to the target tissue for decellularization is accelerated and improved by the use of a vacuum (Butler et al., 2017).

Supercritical fluid: After initial decellularization using detergents like alcohol, supercritical fluid can be employed as a neutralizing agent to remove cellular debris from the tissue since it eliminates it as it travels through the tissue. Supercritical fluid has a fast passage rate and low viscosity, which allows for quick and easy decellularization procedures. The use of an inert material (such as carbon dioxide) to remove cells and little change in the mechanical characteristics of the ECM are two benefits of decellularization using the supercritical fluid approach. The ECM, however, might be obliterated by the pressure needed to introduce the supercritical fluid phase. Additionally, the tissues are dried following the decellularization procedure, doing away with the necessity for lyophilization, a typical method for facilitating long-term preservation (Boer et al., 2015).

Supercritical fluid is permeable like gasses and has the same density as liquids. SC-CO2 has a critical temperature and pressure of 31.1°C and 7.40 MPa, respectively. These values match the physiological circumstances of 37°C and 15 MPa. Additionally, because of the tissue’s great permeability, carbon dioxide gas is promptly expelled from it without the need for extra washing. The polar portion of the membrane, which is a phospholipid, may readily be removed by the addition of ethanol even though carbon dioxide gas is non-polar. Additionally, the tissue’s whole mechanical and structural characteristics are unaltered (Gilpin and Yang, 2017).

One of the useful methods of decellularization is immersion and the induction of turbulence, which allows chemicals to reach tissue cells more effectively and improves decellularization. This technique is used to decellularize tissues that lack a substantial circulatory network, making it impossible for decellularization agents to directly access every region of the tissue. The bladder, esophagus, trachea, skeletal muscles, tendons, heart valves, and cartilage are just a few examples of these tissues. In this technique, the target tissue or organ is submerged in the decellularization agent-filled chambers. The type of decellularization agent used, the thickness and density of the tissue, and the duration of immersion are all factors (Keane et al., 2015).

Perfusion: One way to decellularize organs is to create flow and perfusion in two opposing directions, fully separating the organ from its primary artery. Additionally, chemicals are pumped into its vascular system after washing with detergents. Controlling the flow rate of fluids is crucial because the pressure needed to push detergents and chemicals along the vascular system can lead to capillaries and tiny arteries rupturing (Crapo et al., 2011).

### Enzymatic techniques

The most popular decellularization techniques involve the use of enzymes, which are frequently combined with chemical techniques to aid in eliminating cells and fully demolishing nuclear material from tissue. By stifling connections between cells and the ECM or by locating and eliminating undesirable proteins, enzymes are employed to decellularize cells. Enzymes including trypsin, protease, lipase, hyaluronidase, dispase, collagenase, papain, and nucleases are utilized in these procedures (Rieder et al., 2004; Pellegata et al., 2013).

One of the most used enzymatic decellularization agents is trypsin. ECM proteins do, however, exhibit some degree of resistance to trypsin, therefore use caution when exposed to it. Trypsin eliminates cells more gradually than detergents, damages elastin and collagen more severely, but better retains GAG content. Trypsin usage causes changes in the mechanical characteristics of ECM and decreases its strength by destroying collagen and breaking the connections that hold it together. To disrupt the tissue’s architecture and provide the potential of quick dissolving by detergents or enzymatic chelating agents, trypsin is an aggressive enzyme that particularly cuts peptide chains from the carboxyl side of the amino acids lysine or arginine. Rarely is trypsin utilized as the primary decellularization agent. Trypsin concentrations of 0.05–0.2% (w/v) are often only used during the first stage of therapy, before chemical decellularization. The biomechanics of the ECM can be harmed by trypsin at higher doses or after prolonged exposure (Olsen et al., 2004; Giraldo-Gomez et al., 2016).

Pepsin: Pepsin is one of the key components used to create hydrogels from decellularized tissues for the creation of therapeutic therapies and *in vitro* tissue ECM modeling. ECM of lyophilized tissue is dissolved in an acidic buffer using pepsin. This procedure involves dissolving Decellularized ECM (dECM) powder in a monomeric suspension at room temperature, neutralizing the pH, and then preparing a heat-sensitive pre-gel solution that will polymerize into ECM hydrogel when incubated at 37°C (Fruton, 1970).

Collagenase: Collagenase is a powerful enzyme that breaks down collagen II in cartilage and collagen I and III in other tissues during tissue decellularization. Because of the high abundance of collagen in these samples, collagenase is crucial for decellularization in studies intended to investigate ECM proteins by proteomic methods. Treatment with it can be used for the selective metabolism of ECM collagens, allowing the identification of proteins that control cell function in low-abundance decellularization scaffolds (Kuljanin et al., 2017).

Phospholipase: Phospholipase targets lipids and hydrolyzes ester bonds; it is useful for fatty tissues. But often, it is insufficient to eliminate all lipids when used alone. Phospholipase A2, an esterase, hydrolyzes the phospholipids in the cell but leaves intact the collagen and proteoglycan content. It also significantly reduces the amount of glycosaminoglycan while causing modest structural damage to the ECM. To produce an appropriate decellularized ECM, phospholipase is typically employed to remove cellular material from tissue in conjunction with chemical detergent or non-detergent techniques (Wu et al., 2009). Dispase is a neutral protease that swiftly divides fibronectin and collagen IV to release cells from tissue. This is therefore perfect for decellularizing tissues since collagen IV and laminin make up most of the basement membrane. Dispase can also be used to stop cellular aggregation (Prasertsung et al., 2008; Gonzalez-Andrades et al., 2011).

Nucleases are enzymes that can dissociate the phosphodiester linkages that hold single-stranded and double-stranded nucleic acids’ nucleotides together. In post-treatment procedures for chemical, physical, or biological decellularization, nucleases like RNase and DNase are frequently utilized. To effectively remove considerable amounts of cellular material from the matrix, these agents fracture the nucleus’ contents (Liao et al., 2007).

Following treatment with decellularization chemicals that cause cell lysis, DNase and RNase are very helpful for eliminating nucleotides from the ECM. According to studies, adding DNase treatment to various decellularization tactics based on chemical, enzymatic, and physical approaches can help preserve the biomechanical and glycosaminoglycan characteristics. ECM that has been exposed to DNase or RNase treatment has to go through several washing cycles since these enzymes generate immunogenicity that can thwart recellularization (Rieder et al., 2004).

**Figure 4:**
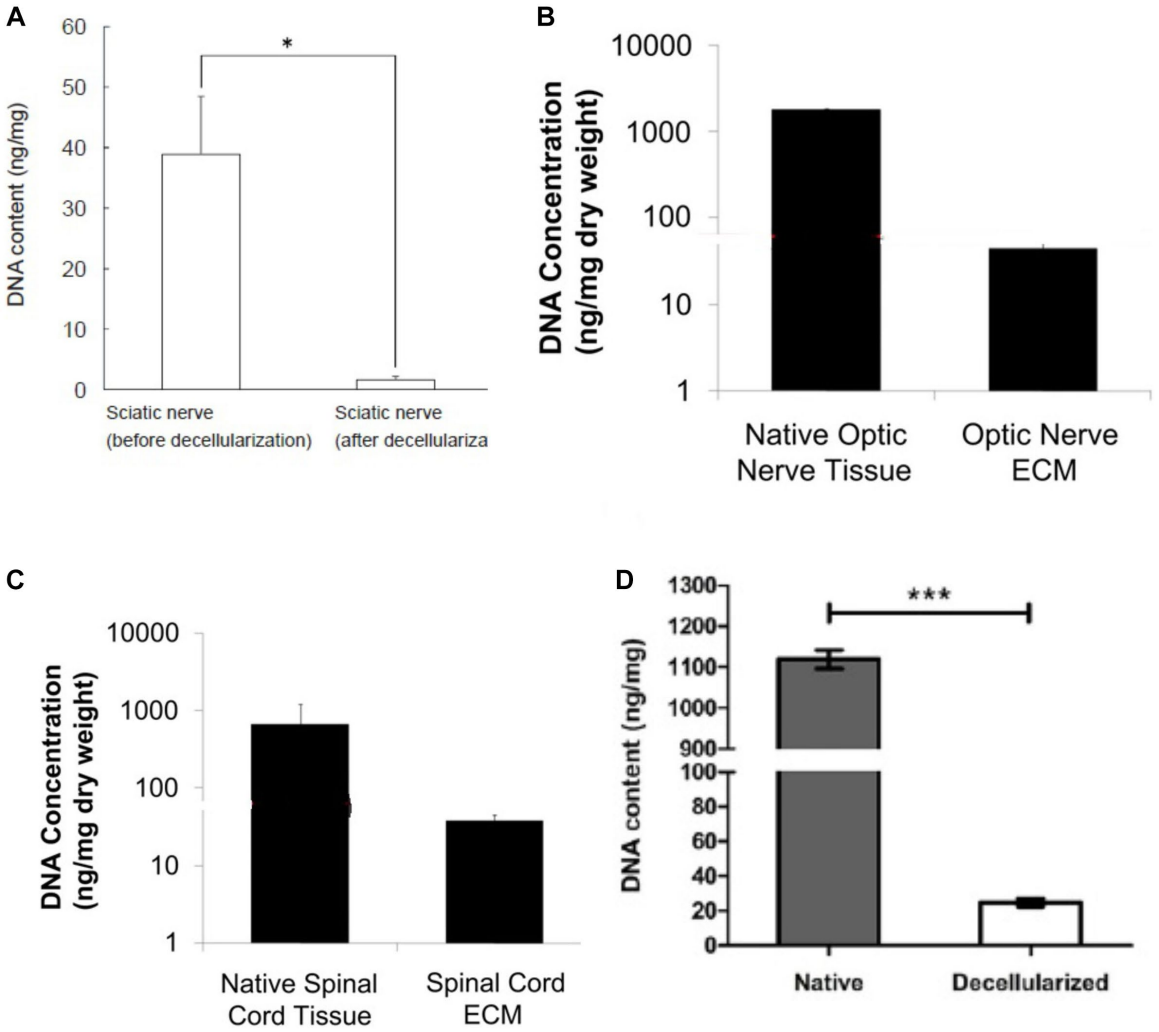
DNA content in DNT by different methods, **(A)** decellularization of pig sciatic nerve with ethanol and 0.5 NaOH solution (Choi et al., 2018), **(B,C)** decellularized porcine optic nerve and spinal cord using 0.02% trypsin/0.05% EDTA (w/v), 4.0% SDC (w/v) and 0.1% peracetic acid (vol) (Crapo et al., 2012), **(D)** decellularized porcine sciatic nerve using 4% Triton X-100 (w/v) (Kong et al., 2022b). In all the methods, the amount of DNA has been greatly reduced compared to the native tissue. Adapted and reprinted.

Chondroitinase is excellent for decellularizing thick cartilage tissue and may aggressively break down proteoglycans. The dramatic decrease in glycosaminoglycan content, which changes the mechanical characteristics of the ECM and makes it stiffer, is directly connected to chondroitinase (Natoli et al., 2009; Bautista et al., 2016). The content of residual DNA in different neural tissues is different depending on the method of cell removal. It should be noted to choose a method that causes the least damage to the ECM structure and on the other hand, cell removal is done successfully. As can be seen in Figure 4, nerve tissues from different sources such as sciatic, spinal, and optic have been decellularized by different methods. The DNA content in all tissues is significantly reduced, but as can be seen in Figures 4B,C, where decellularization was performed with several different detergents, the DNA content is greatly reduced compared to the protocols that One or two substances that have been used in decellularization. A list of the key methods employed in the decellularization process is provided in Table 2.

**Table 2:** The most crucial methods employed in th decellularization process.

Reagent	Reagent type	Advantages and disadvantages
SDS	Chemical (ionic)	This reagent removes cellular components, preserves collagen, glycoproteins, and fiber orientation in tissues, but its strong protein affinity disrupts interactions and collagen integrity, affecting the extracellular matrix and signaling.
SDC	Chemical (ionic)	SDC is advantageous for removing cells and DNA from tissues and organs, but it can also significantly disrupt cellular structures, which is a drawback.
Triton x-100	Chemical (non-ionic)	Triton X-100 is a powerful detergent used for degreasing, effectively removing cells from the ECM surface. However, its impact on tissue structure can vary depending on the tissue’s composition and design.
CHAPS	Chemical (bipolar)	CHAPS is effective for delicate tissue, but its efficiency in thick tissues remains a matter of debate.
NaoH	Chemical (bases)	This reagent efficiently removes cell contents but weakens the ECM and damages collagen.
Peracetic acid	Chemical (acidic)	PAA effectively sterilizes and oxidizes microbial enzymes but can denature ECM proteins like GAGs and collagen, weakening the ECM.
Acetone	Chemical (alcohols)	Acetone extracts lipids in decellularization but does not effectively preserve tissues.
Heat shock	Physical	Thermal shock effectively removes cells but damages cell membranes due to ice crystal formation.
Hydrostatic pressure	Physical	Hydrostatic pressure accelerates cell elimination but can harm the ECM due to ice crystal formation.
Supercritical fluid	Physical	This method removes cells with inert materials but damages the ECM due to pressure constraints.
ultrasonic waves	Physical	This method of tissue decellularization has both benefits and drawbacks concerning tissue structure and mechanical characteristics.
Trypsin	Enzymatic	Using trypsin to enhance decellularization can have both benefits and drawbacks, including slow cell removal, damage to collagen and elastin, and weakening of the ECM.
Pepsin	Enzymatic	Pepsin is vital for creating hydrogels from decellularized tissues but significantly decreases DNA content.
Collagenase	Enzymatic	This technology can extract natural collagen but weakens mechanical strength and damages the ECM structure.
Nuclease	Enzymatic	This method can completely eliminate organ nuclei but is compatible with various types of decellularization agents.

## Protocols of nerve decellularization

The primary purpose of decellularization is to remove all cell components, such as myelin and Schwann cells, from the functional structure of the basal lamina and ECM. Aggressive decellularization protocols aim to reduce immunogenicity to utilize the tissue as a neural scaffold (Nakamoto et al., 2021). Various combined protocols involve enzymatic approaches to remove cellular parts from the ECM, followed by the dissolution of cytoplasmic and nuclear components using detergents (Gilbert et al., 2006).

Table 3 presents the most common protocols used in nerve tissue decellularization. One significant protocol is Sondell’s nerve, which involves soaking the nerves in distilled water for 7 h, followed by a shift to a mixture of distilled water and 3% Triton X-100 (w/v) or one night, and finally placing them in a mixture of 4% SDC (w/v) and distilled water for 24 h. After several repetitions at room temperature, the nerves are washed with distilled water and stored in phosphate-buffered saline (PBS) at 4°C (Philips et al., 2018).

**Table 3:** The most common protocols used in nerve decellularization.

Donator	Tissue source	PNS/CNS	Method	References
Rat	Sciatic	PNS	SD and Triton X-100, 24 h	Sondell et al. (1998)
Rat	Sciatic	PNS	0.14% Triton X-200 (w/v), 0.6 mM SB-16, 10 mM phosphate, and 50 mM sodium and Sondell, 24 h	Hudson et al. (2004b)
Rat	Sciatic	PNS	Freeze, 4 min Treatment with chondroitinase, 16 h	Krekoski et al. (2001)
Rat Rabbit	Sciatic	PNS	4% Triton X-100 (w/v) in ddH2O and agitated in 3% SDC (w/v) in ddH2O, 27 h	Jia et al. (2012)
Rat	Sciatic	PNS	SB-10 and SB-16 with Triton X-200	Chato-Astrain et al. (2020)
Rat	Sciatic	PNS	Sondell, 24 h	Wachs et al. (2022)
Rat	Sciatic	PNS	Hyperosmolar sodium chloride solution (1 M), shaken at 160 rpm for the nerve was then dipped into PBS and washed for an additional at 160 rpm with shaking, 8 days	Ishida et al. (2014)
Cadaver	Median and sural	PNS	Nonionic and anionic detergent (Triton X-100 and SD), amphoteric detergent, and nuclease (CHAPS, deoxyribonuclease I, and ribonuclease A), 12 h	Bae et al. (2021)
Rat		PNS	Hudson’s protocol (the nonionic detergents Tween 20, Tween 80, and Triton X100; (2) the amphoteric detergents Empigen BB (zwitterionic detergent), SB-10, SB-16, and CHAPS; (3) the anionic detergents Triton X-200, dodecyl benzene sulfonate, sodium caprylate, and SD; and (4) CTAB), 24 h	Kim et al. (2016)
Rat	Sciatic	PNS	Hudson’s protocol, 24 h	Hudson et al. (2004a)
Rat	Sciatic	PNS	PBS solution containing 0.6 mM SB-16 and 0.14% Triton X-200 (w/v), 24 h	Moore et al. (2011)
Rat	Brain (PD)	CNS	3.0% Triton X-100 (w/v), 4.0% deoxycholic acid sodium salt (w/v), 0.1% peracetic acid (vol) in 4.0% ethanol (vol), 52 h	Lin et al. (2017)
Rat	Brain	CNS	Water (16 h at 4°C), 0.02% trypsin/0.05% EDTA (60 min at 37°C) (vol), 3.0% Triton X-100 (vol) (60 min), 1.0 M sucrose (15 min), water (15 min), 4.0% SDC (vol) (60 min), 0.1% peracetic acid (vol), in 4.0% ethanol (vol), (120 min), PBS (15 min), water (15 min) and PBS (15 min), 32 h	Crapo et al. (2012)
Rat	Sciatic	PNS	Immersing in sterile pure water (24 h), eluting with 4% Triton X-100 (w/v) (6 h), 4% SDC (w/v) (12 h), rinsing with a mixed solvent (ethanol: dichloromethane = 1:2) and wash several times with PBS, freeze-dring and ground into a powder and stored at 4°C for later use, >42 h	Kong et al. (2022b)
Pig	Sciatic	PNS	Soaking in ethanol with 0.5 N NaOH solution (24 h), neutralizing with 0.5 N HCl, then washing with distilled water, >24 h	Choi et al. (2018)
Rat	Sciatic	PNS	Agitated in deionized water (7 h) and treated in 3% Triton X-100 (w/v), and 4% SDC (w/v) for (24 h), washing in PBS solution carefully, sterilizing by Co60 irradiation (12 h) and stored in PBS solution at 4°C, 43 h	Rao et al. (2021)
Beagle dogs	Sciatic	PNS	Distilled in a mix of water and Triton X-100 for (12 h), deoxycholate (24 h) was washed in PBS, sterilization by Co60 irradiation, and stored in 0.01 M PBS at 4C, >36 h	Qiu et al. (2020)

## CNS regeneration

Tissue engineering offers several approaches to traumatic brain injury (TBI), one of which is ECM decellularization, where the scaffold preserves growth factors and inflammatory factors, promotes neural stem cell transformation, and supports regeneration (Crapo et al., 2012). Another approach involves using brain patches made from decellularized brain tissue to treat injuries (Zhang et al., 2022a). In a specific *in vivo* study targeting Parkinson’s disease (PD) treatment, researchers explored the potential benefits of using bFGF (basic fibroblast growth factor) on rats after performing a complete decellularization protocol and analyzing the tissue through hematoxylin and eosin and DAPI staining. The evaluation of physical response was conducted by counting apomorphine-induced rotations in the rats, and the results showed an improvement in their behavior (Lin et al., 2017). However, despite these efforts, achieving a full recovery remains challenging.

## PNS regeneration

In animal studies, the effectiveness of decellularized nerve transplantation has been demonstrated in preventing muscle atrophy and paresis after nerve transection. Recent studies have shown that Sondell-based protocols for grafts are effective and function well in humans for nerve injuries below 3 cm in length (Song et al., 2017). However, poor outcomes have been reported in animal studies for allografts with diameters longer than 14 mm (Whitlock et al., 2009; Moore et al., 2011).

The sciatic nerve is crucial in peripheral nerve injury (PNI) studies due to its length and easy accessibility for surgery (Geuna, 2015). Emerging from the lumbosacral plexus, it divides into the tibial nerve, the sural nerve, and the common peroneal nerve near the knee (Rupp et al., 2007). dECM can be sourced from nerve tissues like the sciatic nerve in rats and other mammals, as well as from the central nervous system in the context of PD and TBI (Choi et al., 2018; Qiu et al., 2020). These dECM applications aim to decrease immunogenicity, promote regeneration, and achieve functional recovery in tissue defects. In mammalian models like adult beagle dogs, dECM has shown enhancement in regeneration and recovery after autografts, particularly in PNS repair (Qiu et al., 2020) (Table 4).

**Table 4:** Application of DNT in nerve regeneration.

Animal species/Sex	Decellularized tissue/Implantation site	Study period	Finding	References
Rat/Female	Sciatic/Sciatic	About 20 days (Not mentioned exactly)	Become allografts for the repair of the sciatic nerve	Sondell et al. (1998)
Rat/Male	Sciatic/Sciatic	84 days	The decellularization process averted cell-mediated rejection of the grafts	Hudson et al. (2004b)
Rat/Female	Sciatic/Sciatic	8 days	Bloc chondroitinase treatment of acellular nerve grafts effectively degraded CSPG without compromising the basal lamina scaffold or dislocating its laminin content	Krekoski et al. (2001)
Rat& rabbit/Not mentioned	Sciatic/Sciatic	8 weeks	Muscular atrophy and motor function were recovered partially	Jia et al. (2012)
Rat/Female	Sciatic/Sciatic	12 weeks	The percentage of neurotrophic ulcers was significantly higher in the RSN group than in the SD group	Chato-Astrain et al. (2020)
Rat/Not mentioned	Sciatic/Sciatic	11 weeks	The sciatic nerve action potentials, the thickness of the myelin sheath, the diameter of the axon and the dominated muscle are more rehabilitated than the blank group	Wang et al. (2017)
Rat/Male	Sciatic/Sciatic	8 weeks	nearly all functional and histological assessments performed in a rat defect of the sciatic nerve over an 8-week time course	Wachs et al. (2022)
Rat/Female	Sciatic/Sciatic	2 months	Resulting in the generation of a structure that was almost the same as that of the intact nerve.	Ishida et al. (2014)
Rat/Not mentioned	Sciatic/Sciatic	Not mentioned	The optimized protocol is comparable to the Sondell protocol in its ability to remove cellular components and is better at retaining the natural ECM	Hudson et al. (2004a)
Rat/Male	Sciatic/Sciatic	16 weeks	Host nerve tissue distal to the interposed silicone conduit appeared atrophic and translucent, suggesting a general absence of healthy myelinated axons	Moore et al. (2011)
Rats/Not mentioned	Sciatic/Sciatic	12 weeks	The function and response of the thermal motivator of autograft nerves are like normal nerve	Kong et al. (2022b)
Rat/Not mentioned	dcBECM/dcBECM (PD)	2 weeks	Effective in 11 removing the cellular components while keeping the micro- and ultra-structure characterized of the brain architecture and composition of the matrix	Lin et al. (2017)
Pig/Not mentioned	Sciatic/Sciatic	24 weeks	After 18 weeks, displayed statistical differences but at 24 weeks, they were not retained between groups, and Lower self-mutilation in the GFNC group than in others	Choi et al. (2018)
Beagle dog/Not mentioned	Sciatic/Sciatic	6 months	enhance nerve regeneration and functional recovery after repair of long nerve defects	Qiu et al. (2020)

## Advantages of using decellularized tissues in nerve regeneration

The use of decellularized tissues, particularly decellularized nerve tissues, has been the focus of more research since the use of polymers in neural regeneration is sometimes hampered by complications. Decellularized tissues have several benefits, including their distinct structure, the presence of bioactive chemicals, relatively subdued immunological responses, excellent biodegradability, and a reduction in the difficulties associated with organ replacement (Liao et al., 2020). To rebuild peripheral nerve damage, the review of the study data suggests that designed nerve grafts are an appropriate substitute for autologous nerve grafting. The creation of peripheral nerves requires the use of an appropriate scaffold. Because it offers a favorable environment for cell adhesion and growth. Additionally, it can release growth hormones and quicken axon regeneration. Engineered scaffolds can improve regeneration conditions and hasten vascularization in seizures (Gontika et al., 2018; Asadi et al., 2020).

According to reports, the fundamental elements of decellularized matrices, such as collagen, elastin, and laminin, are what enable the aforementioned biological processes (Zheng et al., 2014; Liao et al., 2020). In studying the interactions of the ECM of the PNS, decellularized nerve grafts for the restoration of peripheral nerves can be utilized as a model. The environment for cell growth, reproduction, and migration is favorable when using clamps of this sort as a physical scaffold. In fact, by lowering antigenicity, these grafts can be a viable therapeutic approach (particularly when autologous tissues are not accessible) (Abbaszadeh et al., 2020). The immune system’s actions can be modulated by decellularized tissues, which have been shown to have limited immunogenicity due to their generally weak immunological responses (Brown et al., 2015). After decellularization, the majority of cells, antigen components, and other substances are typically eliminated, which reduces the immunogenicity of the decellularized tissues (Valentin et al., 2009; Yu et al., 2022). According to studies, the removal of cells and other antigen-containing elements from natural tissues can result in porous structures with the proper width, which can subsequently improve nutrition exchange while promoting adhesion and cell proliferation. Decellularized tissues are said to be capable of being crucial to the process of regenerating tissues and organs. They aid in tissue regeneration since they not only control the healing process but also when the repair is complete, are spontaneously eliminated (Liao et al., 2020). Decellularized scaffolds, a special structure made up of ECM structural proteins, growth factors, hormones, and other bioactive substances, can actually encourage repair and regeneration after nerve loss by creating a receptive environment (Buckenmeyer et al., 2020). The application of this method holds promise for the production of biologically active compounds that could promote the restoration of the nervous system’s functionality specifically (Maghsoudlou et al., 2013; Meng et al., 2014).

Studies show that the internal structural and molecular components of the extracellular matrix are preserved in decellularized nerve grafts, supporting neural regeneration (Szynkaruk et al., 2013). Many rat, mouse, and sheep models have been used to study the immune characteristics of cold-preserved allografts. These findings indicate a gradual loss of immunogenicity (Fox and Mackinnon, 2007). While the CNS has a limited capacity for regeneration, the spinal cord has shown more promise when using decellularized components. According to studies, using decellularized ECM scaffolds can influence host cell phenotypes, differentiation, migration, proliferation, and other aspects that can aid in the regeneration of CNS tissues. Additionally, it has been demonstrated that dECM-derived scaffolds can serve as a guiding substrate and a transporter of sequestered bioactive substances to support proper axonal sprouting (Guo et al., 2010; Xu et al., 2016). The evidence suggests that the decellularized allograft can improve motor and sensory function in relatively small nerve gaps (approximately 2.3 cm) clinically (Tang and Chauhan, 2015).

## Challenges, limitations, and future prospects of using decellularized tissues in nerve regeneration

While some preclinical and clinical studies have shown encouraging results using decellularized matrices, there are several technical and grafting challenges in the clinical use of dECM scaffolds. For example, there have been reports of non-infectious edema, severe pain, and inflammatory response from decellularized scaffolds. Evidence also suggests that acellular scaffolds may not be suitable for injuries requiring long-term mechanical support. Furthermore, it has been reported that dECM scaffold implantation may lead to scar tissue deposition without net tissue regeneration (Elmashhady et al., 2017). The evidence indicates that the most common methods of decellularization of nerve tissues include perfusion, chemical, and biological agents, which are often used in combination with a physical method. The mentioned methods may change or destroy the important components of ECM, which in turn affects the structure, adhesion, and proliferation of future cells (Uriarte et al., 2018).

For example, treatment with acidic or alkaline solutions and ionic detergents may dissolve acidic components and disrupt the structure of nucleic acids. Non-ionic detergents may also leave protein–protein interactions intact and this is not desirable. The use of hypertonic and hypotonic solutions may also lead to osmotic shock and eventually cell lysis. The use of enzymes, in turn, may change the stability of the ECM due to the change in collagen content (Arslantunali et al., 2014).

Preservation of ECM microarchitecture and composition during decellularization requires optimal protocols that provide efficient removal of cells with minimal disruption. It has been reported that the balance between the effective removal of cells and preservation of structure and biochemical and biomechanical properties to obtain a dECM scaffold that minimizes immunogenicity after the implantation process and provides an optimal balance between cells and ECM is challenging. The evidence indicates that inefficient decellularization by the mentioned substances can cause immune system rejection after *in vivo* implantation. Another challenge is the effective and optimal cellularization of the nerve and determining the number of cells needed for the optimal cellularization process (Barbulescu et al., 2022).

The decellularization of tissues and organs to create ECM bioscaffolds, according to studies, necessitates a delicate balancing act between preserving the ECM’s structure and removing cellular components such as DNA, mitochondria, membrane lipids, and cytosolic proteins. These cellular remnants can cause an unfavorable inflammatory reaction and prevent the healthy regeneration of the tissue if they are not properly eliminated (Keane et al., 2015; Kubinova, 2017; Liao et al., 2020). Therefore, care should be taken to minimize the presence of normal cells that can trigger negative immunological reactions during the decellularization of an organ. According to studies, insufficient decellularization can hinder recellularization, which in turn causes graft rejection (Keane et al., 2015). The low mechanical stability of models based on decellularized ECM is one of their main drawbacks. The mechanical characteristics of decellularized tissues frequently differ significantly from those of their native equivalents, which may limit the scaffold’s capacity to hold its shape *in vitro* and modify cellular behavior (Lovati et al., 2018; Barbulescu et al., 2022). Utilizing natural and synthetic polymers, such as polycaprolactone, silk fibroin, polylactic-co-glycolic acid, chitosan, hyaluronic acid, alginate, and polyethylene glycol, is one way to solve this problem. Utilizing cross-linkers such as glutaraldehyde, riboflavin/ultraviolet light, genipin, and carbodiimide is another technique to enhance the mechanical characteristics (McCrary et al., 2020).

The re-endothelialization of decellularized tissue is the next difficult task. There should be more efforts to preserve ECM structure and endothelialization (e.g., physical, enzymatic, and chemical means), as research has shown that variations in the microstructure and quantity of ECM components (e.g., GAGs, elastin, and fibronectin) might affect re-endothelialization. The density of the target organ, its fat content and thickness, the type of decellularization agent, their concentration, solution pH, temperature, and decellularization duration should all be taken into account throughout the decellularization process (Mazloomnejad et al., 2023).

Artificial and biological bioreactors have produced endothelialized acellular vasculature in the field of recellularization, but full endothelialization has not yet been accomplished. The issues that arise from transplanting acellular structures and the lack of flow rate, pressure, and shear stress parameters for each organ in artificial bioreactors should be completely addressed in future studies. Another important factor in the endothelialization of acellular structures that was studied was surface alteration. To enhance ECM structure and endothelialization levels, adhesive molecules and GFs have been employed (Ye et al., 2013; Marinval et al., 2018).

The transition of these investigations into human clinical trials remains a top priority notwithstanding the encouraging results. Alternative strategies should also be investigated to enhance re-endothelialization outcomes. For comprehensive endothelium coverage, exosomes—extracellular vesicles carrying RNA, DNA, proteins, and lipids—might be useful. For their efficacy to be confirmed, more research is required (Nazerian et al., 2021). Another challenge is the effective and optimal cellularization of the nerve and determining the number of cells needed for the optimal cellularization process (Huang et al., 2017; Barbulescu et al., 2022). According to reports, even though the use of dECM may offer a different treatment to promote peripheral nerve regeneration, the antigenic material transmitted from allograft specimens during this process mandates the use of immunosuppressants and requires surgery (Buckenmeyer et al., 2020). Therefore, based on the information provided, it is highly advised to carry out additional research that will aid in resolving the issues raised. Combining decellularized nerve tissue with other methods, such as stem cell transplantation, provides a better perspective on the potential for nerve repair in the future. Also, the use of such decellularized tissues with polymers can be effective in creating appropriate porosity and preventing microbial contamination in the transplant. In summary, these methods have the potential to become a valuable choice for the regeneration of damaged central and peripheral nerves and can bring promising products to the market (Hopf et al., 2022; Kong et al., 2022a).

## Conclusion

Decellularized tissues have the innate capacity to promote tissue regeneration, making them valuable in various disease models and therapies. Decellularization can be achieved through mechanical, chemical, or enzyme exposure techniques. The resulting acellular scaffolds contain growth factors, hormones, bioactive compounds, and essential proteins from the ECM, creating a favorable environment for repair and regeneration after injury. This method holds the potential to create physiologically active compounds that aid in the functional recovery of the nervous system. Previous attempts to promote axonal elongation using growth factors and biomaterial implants have shown limitations, resulting in random and abnormal axonal development, or limited functional outcomes. Decellularized tissue-derived scaffolds act as guiding substrates and delivery systems for isolated bioactive substances, encouraging healthy axon sprouting. Utilizing decellularization to create cell-free neural transplants eliminates donor-site complications associated with using neural autografts and reduces the need for post-surgical immunosuppression by removing antigenic material from allografts. The selection of appropriate decellularization techniques for tissues is crucial in facilitating nerve defect healing, as emphasized by this research.

## Author contributions

MM: Writing – review & editing. TST: Writing – review & editing. ZA: Writing – review & editing. LR: Supervision, Writing – review & editing. MK: Writing – review & editing.

## Figures and Tables

**Table:** 

3D	Three dimensional
AD	Alzheimer’s disease
Aβ	Amyloid β
CHAPS	(3-cholamidopropyl) (dimethylammonio]-1-propane sulfonate
CMC	Carboxymethyl chitosan
CNS	Central nervous system
CNT	Carbon nanotubes
CSPG	Chondroitin sulfate proteoglycans
DAPI	diamidino-2-phenylindole
ddH2O	*Double-distilled water*
dECM	Decellularized ECM
DNT	Decellularized nerve tissues
ECM	Extracellular matrix
EDTA	ethylenediaminetetraacetic acid
EGTA	ethylene glycol-bis (β-aminoethyl ether)-N, N,N′,N′-tetraacetic acid
ESCs	embryonic stem cells
FDA	Food and Drug Administration
FGF-b	basic fibroblast growth factor
GAG	Glycosaminoglycan
GCS	Glasgow Coma Scale
GDNF	Glial-derived neurotrophic factor
H&E	Hematoxylin and eosin
HA	Hyaluronic acid
iPSCs	Induced pluripotent stem cells
NaoH	Sodium hydroxide
NFT	Neurofibrillary tangles
NMDA	N-methyl-D-aspartate
NSCs	Neural stem cells
OPF	Oligo-polyethylene glycol fumarate
PAA	Peracetic acid
PANI	Polyaniline
PBS	Phosphate-buffered saline
PCL	Poly caprolactone
PCLF	Polycaprolactone fumarate
PD	Parkinson’s disease
PEDOT	Polyethylene 3 and 4 dioxythiophene
PEEK	Polyether Ether Ketone
PGA	*Poly glycolic acid*
PGA	Poly-L-lactic glycolic acid
PHAS	Polyhydroxyalkanoate
PHB	Poly 3 hydroxybutyrate
PHBV	Poly-3 hydroxybutyric acid-co-3 hydroxyalric acid
PHBV	Polyhydroxybutyrate valerate
PLA	Polylactic acid
PLCL	l-lactide-co-ε-caprolactone
PLGA	Polylactic -co-glycolic acid
PLLA	Poly-L-lactic *acid*
PNS	Peripheral nervous system
PPY	Polypyrrole
PU	Polyurethane
PVA	Polyvinyl alcohol
RPM	Revolutions per minute
RT	Room temperature
SB-10	Sulfobetaine 10
SB-16	Sulfobetaine 16
SC-CO2	Supercritical carbon dioxide
SCI	Spinal cord injury
SDC	Sodium deoxycholate
SDS	Sodium dodecyl sulfate
SF	Silk fibroin
SNpc	Substantial nigra pars compacta
TBI	Traumatic brain injury
TGF-β	Transforming growth factors β
VEGF	Vascular endothelial growth factor
WD	Wallerian degeneration
